# External Carotid Artery Entrapment by the Hyoid Bone Associated with an Atherosclerotic Stenosis of the Internal Carotid Artery

**DOI:** 10.3390/diseases12100258

**Published:** 2024-10-18

**Authors:** Grigol Keshelava, Zurab Robakidze, Devi Tsiklauri

**Affiliations:** Department of Vascular Surgery, Clinic Healthycore, 0112 Tbilisi, Georgia; zurab.robakidze@yahoo.com (Z.R.); doc.tsiklauri@gmail.com (D.T.)

**Keywords:** external carotid artery entrapment, carotid endarterectomy, greater horn of hyoid bone, hyoid bone resection

## Abstract

The mechanical compression of an external carotid artery (ECA) is a rare pathology. The compression of the carotid bifurcation can be positional, induced by anatomical elements, or provoked by volumetric formation in the neck area. In this study, we describe a rare case of an entrapment of the ECA. A 67-year-old man who had two episodes of transient ischemic attack (TIA) demonstrated by loss of consciousness was transferred to our hospital. Ultrasonography and computed tomography revealed the atherosclerotic stenosis (80%) of a right internal carotid artery (ICA) and, at the same time, entrapment of the right ECA by the elongated right greater horn of the hyoid bone (GHHB). A 1 cm section of the GHHB was resected. After clamping of the carotid arteries, longitudinal arteriotomy and endarterectomy surgeries were performed from the right ICA. At the two months follow-up examination, the patient’s condition was reported as normal, with no episodes of TIA, dysphagia, or pharyngeal discomfort.

## 1. Introduction

The mechanical compression of the carotid bifurcation is a rare pathology. The compression can be positional [1], induced by the anatomical elements [2], or provoked by volumetric formation in the neck area [3]. In the case of all three types of compression, there is a risk of ischemic stroke, carotid dissection, and rupture [4,5,6]. 

Anatomical elements, such as an elongated styloid process or elongated GHHB, may cause carotid vessel compression. The hyoid bone (HB) is also close to the carotid vessels, and variations in anatomy can cause carotid vessel compression, stenosis, dissection, or pseudoaneurysm formation [4]. Carotid artery entrapment by the HB is a rare phenomenon that is relatively unknown to clinicians. 

The HB, a horseshoe-shaped bone that forms attachments with several muscles, helps with tongue movement and swallowing. It is located between the thyroid cartilage and the mandible in the anterior region of the neck, usually at the level of the C3 vertebra. The HB consists of a body and two pairs of protrusions, called the greater and the lesser horns. An elongation of the greater horn of the hyoid bone occurs during embryonic development [7]. Therefore, a basic knowledge of HB embryology enables an understanding of the etiology of various anatomical variants. In the general population, anatomical variations in the HB occur between 4% and 30% of the time [8], but a GHHB that is too long rarely compresses the carotid vessels [5]. Therefore, because of the scarcity of reported cases, the optimal treatment is unclear. In the literature, both medical and surgical treatments are described [2,9,10,11].

In this study, we report an unusual case of the right ECA entrapment and occlusion by the elongated GHHB associated with symptomatic atherosclerotic stenosis of the ipsilateral ICA. One-stage GHHB resection and carotid endarterectomy were successful in this case. 

## 2. Case Presentation

**Figure 1:**
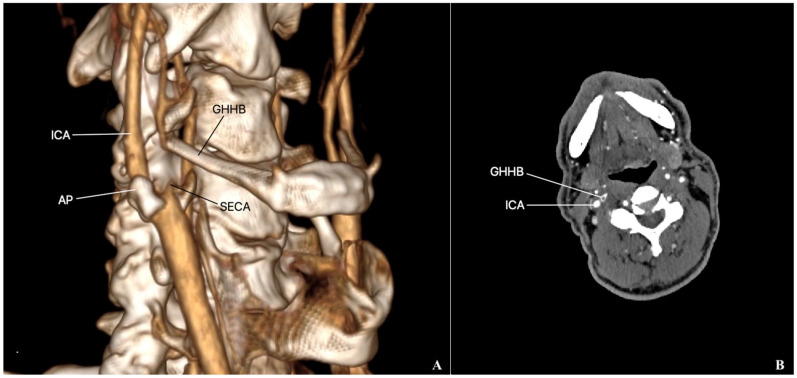
(**A**) CTA reveals the atherosclerotic stenosis of the right ICA and the entrapment of the right ECA by the GHHB. (**B**) Axial view of the carotid bifurcation. ICA: internal carotid artery; AP: atherosclerotic plaque; GHHB: greater horn of the hyoid bone; and SECA: stump of external carotid artery.

Our hospital received a 67-year-old man who had two episodes of TIA, characterized by loss of consciousness. The interval between the two episodes was one month. Risk factors included hypertension, hyperlipidemia, and tobacco abuse. At the time of admission, there were no neurological sequelae. Computed tomography angiography (CTA) revealed the atherosclerotic stenosis (80%) of the right ICA and, at the same time, an entrapment and occlusion of the right ECA by the elongated GHHB (Figure 1). The left carotid bifurcation was not in contact with the left GHHB. The stenosis of the left ICA reached 20%. The lengths of the right and left GHHBs were 3.2 cm and 2.9 cm, respectively. No stroke foci were detected in the right half of the brain. The patient also underwent an ultrasonography that confirmed 80% stenosis of the ICA and the contact of a GHHB with carotid bifurcation. There was no antegrade blood flow in the ECA. The hemodynamics of an ECA did not change with neck rotation. The intermittent claudication in the region of the masseter muscles on mastication, which could accompany ECA occlusion, was not observed in the patient. In order to prevent stroke and restore blood circulation in the ECA, carotid endarterectomy and GHHB resection procedures were planned. 

**Figure 2:**
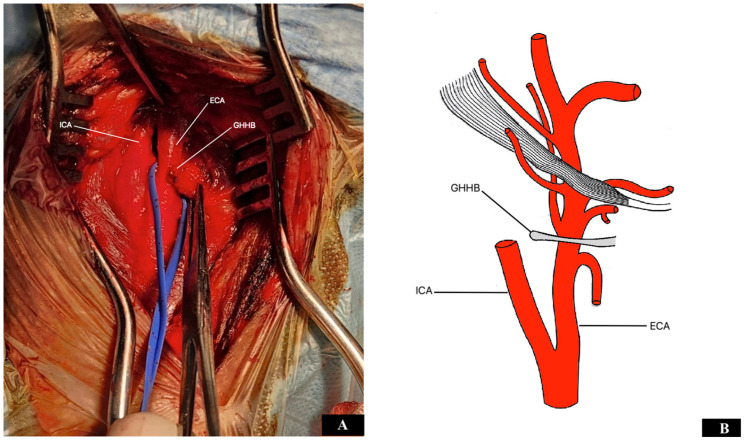
(**A**) Intraoperative photo: surgical exposure of the right carotid arteries and the GHHB. (**B**) Graphical image shows the relationship between the carotid arteries and the GHHB. ICA: internal carotid artery; GHHB: greater horn of the hyoid bone; and ECA: external carotid artery.

The intervention was performed on the patient under general anesthesia. The great saphenous vein in the right thigh was marked for harvesting if needed. The carotid bifurcation and GHHB required a right pre-sternocleidomastoid cervicotomy (Figure 2). After skin and platysma incision and retraction of the sternocleidomastoid muscle, the carotid arteries were exposed as standard. The upper limit of the approach was represented by the digastric muscle and hypoglossal nerve. During the surgical procedure, the external compression of the ECA by the GHHB was confirmed. There was no evidence of arterial perforation or any presence of a pseudoaneurysm; only evidence of contact between both structures was observed. The dissection of connective tissue around the GHHB was performed along the superior, lateral, and inferior borders, being careful not to injure the superior laryngeal nerve. A 1 cm section of the elongated greater horn was resected using a bone cutter. As a result, when the neck rotated, the stump of the GHHB no longer came into contact with the ECA. At the next stage, after systemic heparinization (100 IU/kg) and clamping of the carotid arteries, longitudinal ICA arteriotomy and endarterectomy surgeries were performed. Moreover, a thrombotic mass was removed from the origin of the right ECA. In our opinion, the cause of the formation of a thrombus in the ECA was the external compression on the artery by the elongated GHHB. The atherosclerotic plaque did not spread into the ECA. The arteriotomy was closed with a synthetic patch angioplasty. Carotid endarterectomy and closure of the arterial wall were carried out with the help of a temporary shunt. The operation lasted two hours. The amount of blood loss during the surgical intervention was about 100 mL. The pathological examination confirmed the atheromatous nature of the plaque from the carotid bifurcation. After surgery, the patient was able to follow all commands well and was transferred to the recovery room in stable condition. 

Neurological symptomatology and dysphagia were not detected in the postoperative period. Pharmacological support was instituted according to the hemodynamic situation. Low-molecular weight heparin therapy was started on postoperative day one and continued for one week. Acetylsalicylic acid was used as a permanent antiplatelet therapy in an amount of 100 mg daily. The patient was discharged on postoperative day three and was prescribed daily treatment with clopidogrel, 75 mg; acetylsalicylic acid, 100 mg; rosuvastatin, 40 mg; and calcium channel blocker, 10 mg. At the two months follow-up examination, the patient’s condition was reported as normal, with no episodes of TIA, dysphagia, or pharyngeal discomfort. Ultrasonography revealed patent right common, internal, and external carotid arteries. 

## 3. Discussion 

During the embryonic period, the second and third pharyngeal arch cartilages contribute to the formation of the styloid process, as do the lesser and greater horns of the hyoid bone. The hyoid bone body originates from the hyoid body anlage, with no overt contributions from the second and third pharyngeal arch cartilages [12]. Embryonic development can explain the etiology of an anatomical variation in the HB. The HB stands apart from other bones in the body. It connects to numerous muscles that support and move the tongue, larynx, and pharynx during swallowing and speech. Ligaments attach the ends of the GHHB to the larynx’s hyoid cartilage, whereas the styloid process attaches the lesser horns [13]. During the pharyngeal phase of swallowing, the suprahyoid muscles contract, and the hyoid bone moves forward under the tongue’s base. The HB begins to ossify at the end of the third trimester and may never fully fuse until late in life [14]. 

Typically, the carotid arteries are located posterolateral to the HB and do not come into contact with it. The carotid–hyoid relationship, on the other hand, should have a certain degree of variability over time. Duan et al. studied 74 intact HB and found that the length of a right GHHB was 31.4 ± 2.6 mm and the length of a left GHHB was 31.0 ± 2.5 mm [15]. The GHHB elongation can cause carotid artery entrapment, resulting in dissection, stenosis, pressure-induced plaque formation, or rupture [2]. Manta et al. identified 12 types of the carotid–hyoid relationships: Type I (ECA medial to the GHHB) was presented in 0.34%, Type II (ICA medial to the GHHB) in 0.34%, Type III (ECA and ICA medial to the GHHB) in 1.02%, Type IV (common carotid artery medial (CCA) to the GHHB) in 1.02%, Type V (carotid bifurcation medial to the GHHB) in 0.34%, Type VI (ECA lateral to the GHHB) in 20.41%, Type VIII (ECA and ICA lateral to the GHHB) in 3.74%, Type IX (CCA lateral to the GHHB) in 8.5%, Type X (carotid bifurcation lateral to the GHHB) in 6.46%, Type XI (ICA medial and ECA lateral to the GHHB) in 0.34%, and Type XII (ECA medial and ICA lateral to the GHHB) in 0.34% [16]. 

All cases in the existing literature present compression of the ICA by the elongated GHHB [5,6,9,10,17,18,19,20,21,22]. Only one article described the styloid process compressing the ECA, resulting in pseudoaneurysm formation [23]. We could not find in the literature a case of isolated compression and occlusion of the ECA by the GHHB that was similar to the case described by us. 

It should be noted that in almost half of the cases of compression of the carotid arteries with a GHHB, twisted carotid bifurcation was observed. In all of these cases, the GHHB was situated lateral to the ICA. Conversely, when the ICA ran laterally (normal bifurcation), the GHHB positioned itself medial to the ICA. This result suggests a relationship between the lateral positioning of the GHBB and the twisted carotid bifurcation, although the cause of this relationship remains unknown [21]. Furthermore, reports indicate that certain positional anomalies can undergo reversible changes. Some studies have reported that abnormal ICA positions became normal, or conversely, normal positioning became abnormal during the follow-up period [9,21]. According to these reports, there are cases in which the HB and carotid bifurcation are more flexible than expected, and stroke due to mechanical stimulation by the HB may be more common. In the case described in this study, both carotid bifurcations were in a normal position.

The diagnosis of compression of the ICA is based on ultrasonography and CTA [15]. Some authors prefer an MRI for confirmation of the diagnosis of strokes, such as pseudo-strokes, which may present with similar symptoms [9]. Ultrasonography, using provocative maneuvers with neutral and rotated neck positioning, can confirm the entrapment of carotid vessels. Ultrasonography can reveal changes in flow with positioning [24]. CTA may further characterize the anatomical relationships between the carotid arteries and the HB, but this has the disadvantages of radiation and not being a dynamic study [5,6].

Because of the scarcity of reported cases, the optimal treatment is unclear. Some authors choose to start treatment with anticoagulation and/or antiplatelet therapy [2,10,11]; however, partial GHHB resection, which proved to be effective, remains a viable option. Martinelli et al. note that endovascular intervention and the use of a stent are not advisable; rather, surgery to eliminate the injuring bone compression is recommended [20]. However, some authors describe transient dysphagia in the postoperative period [17]. According to Liu et al., hyoid bone resection may not always be necessary, and a longer-term follow-up period with surveillance imaging would help explore this rare phenomenon [19]. Considering anatomy, the HGGB has attached muscle groups that act during swallowing; therefore, massive resection of these structures may cause dysphagia. Additionally, we must pay close attention to the superior thyroid artery and vein, as damage to these vessels can result in cervical hematoma, and injury to the superior laryngeal nerve can lead to weakness, tightness, and increased speaking effort [21].

In some cases, this pathology is associated with atherosclerotic stenosis of the carotid bifurcation [10]. De Campos et al. stated that trapping the carotid bifurcation can lead to turbulent flow, shear stress, damage to the elastic layer, and the formation of atherosclerotic plaques [25]. Abdelaziz et al. share the same opinion. They speculate on how the HB compression may contribute to the pathogenesis of atherosclerotic carotid artery stenosis. It is thought that when an HB squeezes blood vessels, it might change the way the arterial wall shear forces are built, which could lead to pressure-induced intramural mechanical stress. After that, damage to the endothelium, the buildup of blood components, and the thickening of the myointima occur as the first steps in the atherogenesis cascade and lead to arterial stenosis [5]. However, in the present case, the atherosclerotic plaque extended to the ICA. In the ECA’s compressed region, there was no atherosclerotic process. 

There are two hypotheses regarding the etiology of the TCB. The congenital hypothesis proposes that TCB is a result of excessive mediolateral migration of the ECA during embryogenesis [26,27]. The other hypothesis proposes that elongation and tortuosity of the carotid arteries are due to age-related atherosclerosis [28,29]. 

The ICA appears during the 3 mm embryonic stage (24 days) from the combination of the third branchial arteries and the distal segments of the paired dorsal aortae. The ventral portion of the second branchial arch disconnects from the dorsal aorta and becomes the ventral pharyngeal artery. Eventually, the ventral pharyngeal artery and the ICA fuse proximally to form the common carotid artery and the distal segment of the ventral pharyngeal artery becomes the ECA [30,31]. Any anatomical variations of ICA may arise as result of embryological abnormalities [32]. S- or C-shaped, as well as tortuosity, kinking, and loops of ICA, are believed to be congenital anomalies [33]. The origin of these variations can be understood through the embryological development of the branchial arteries. 

Certain researchers propose that age may continue to be an important factor in the simultaneous occurrence of elongation of the GHHB and TCB. They observe that the GHHB may enlarge over time as a result of joint ossification [34], and similarly, the carotid arteries tend to exhibit increased tortuosity as individuals age [35]. Furthermore, it is essential to take into account the influence of sexual dimorphism, as studies indicate that the distal ends of the GHHB in females are longer compared to those in males [36]. 

In the case described in this study, the patient had two pathologies: symptomatic atherosclerotic stenosis of the ICA and entrapment of the ECA by the GHHB. Therefore, we performed an endarterectomy from the ICA, a GHHB resection, and a thrombectomy from the ECA. It is debatable whether the GHHB resection and thrombectomy from the ECA would be appropriate if there was no symptomatic ICA stenosis. In our opinion, performing a surgical intervention would still be reasonable. The GHHB resection is the prevention of ECA pseudoaneurysm formation [24]. In addition, the ECA creates four important collateral connections with the intracranial arterial system that are of particular importance in the case of ICA occlusion: the anastomoses between the frontal branch of the superficial temporal artery and the lacrimal artery; the anastomoses between the orbital branch of the middle meningeal artery and the lacrimal artery; the anastomoses between the angular branch of the facial artery and the ophthalmic artery; and the anastomoses between the sphenopalatine branch of the maxillary artery and the ethmoidal artery [37].

## 4. Conclusions

The present case examined in this study demonstrates that a one-stage carotid endarterectomy for the symptomatic stenosis of the ICA and a GHHB resection for the ECA entrapment provide good results. However, future studies are necessary to investigate the relationship between an elongated GHHB and the traumatic injury of the internal and external carotid arteries. 

## Data Availability

No new data were created or analyzed in this study. Data sharing is not applicable to this article.

## References

[1] Keshelava G., Nachkepia M., Arabidze G., Janashia G., Beselia K. (2012). Unusual positional compression of the internal carotid artery causes carotid thrombosis and cerebral ischemia. Ann. Vasc. Surg..

[2] Liu G., Wang Y., Chu C., Ren Y., Hua Y., Ji X., Song H. (2021). Hyoid elongation may be a rare cause of recurrent ischemic stroke in youth—A case report and literature review. Front. Neurol..

[3] Keshelava G., Robakidze Z. (2020). Cervical vagal schwannoma causing asymptomatic internal carotid artery compression. Ann. Vasc. Surg..

[4] Schneider C.G., Kortmann H. (2007). Pseudoaneurysm of the common carotid artery due to ongoing trauma from the hyoid bone. J. Vasc. Surg..

[5] Abdelaziz O.S., Ogilvy C.S., Lev M. (1999). Is there a potential role for hyoid bone compression in pathogenesis of carotid artery stenosis?. Surg. Neurol..

[6] Yukawa S., Yamamoto S., Hara H. (2014). Carotid artery dissection associated with an elongated hyoid bone. J. Stroke Cerebrovasc. Dis..

[7] Kopuz C., Ortug G. (2016). Variable morphology of the hyoid bone in Anatolian population: Clinical implications—A cadaveric study. Int. J. Morphol..

[8] De Paz F.J., Rueda C., Barbosa M., Garcia M., Pastor J.F. (2012). Biometry and statistical analysis of the styloid process. Anat. Rec..

[9] Plotkin A., Bartley M.G., Bowser K.E., Yi J.A., Magee G.A. (2019). Carotid artery entrapment by the hyoid bone—A rare cause of recurrent strokes in a young patient. Ann. Vasc. Surg..

[10] Keshelava G., Robakidze Z. (2024). Iternal carotid artery stenosis and entrapment by the hyoid bone. Eur. J. Vasc. Endovasc. Surg..

[11] Ludt C., Leppert M., Jones A., Song J., Kuwayama D., Pastula D.M., Poisson S. (2018). Multiple strokes associated with elongation of the hyoid bone. Neurohospitalist.

[12] de Bakker B.S., de Bakker H.M., Soerdjbalie-Maikoe V., Dikkers F.G. (2018). The development of the human hyoid-larynx complex revisited. Laryngoscope.

[13] Fakhry N., Puymerail L., Michel J., Santini L., Lebreton-Chakour C., Robert D., Giovanni A., Adalian P., Dessi P. (2013). Analysis of hyoid bone using 3D geometric morphometrics: An anatomical study and dissection of potential clinical implications. Dysphagia.

[14] Gupta A., Kohli A., Aggarwal N.K., Banerjee K.K. (2008). Study of age of fusion of hyoid bone. Leg. Med..

[15] Duan K.C., Wang X.R. (1995). Anatomic study on the syndrome of greater-hyoid horn. Chin. Clin. Anat..

[16] Manta M.D., Rusu M.C., Hostiuc S., Vrapciu A.D., Manta B.A., Jianu A.M. (2023). The carotid-hyoid topography is variable. Medicina.

[17] Kolbel T., Holst J., Lindh M., Matzsch T. (2008). Carotid artery entrapment by the hyoid bone. J. Vasc. Surg..

[18] Ye Q., Liu Y., Tang H., Zhou Q., Zhang H., Liu H. (2023). Hyoid bone compression—Induced carotid dissecting aneurysm: A case report. Exp. Ther. Med..

[19] Liu S., Nezami N., Dardik A., Nassiri N. (2020). Hyoid bone impingement contributing to symptomatic atherosclerosis of the carotid bifurcation. J. Vasc. Surg. Cases Innov. Tech..

[20] Martinelli O., Fresilli M., Jabbour J., Di Girolamo A., Irace L. (2019). Internal carotid stenosis associated with compression by hyoid bone. Ann. Vasc. Surg..

[21] Ishikawa T., Moribe K., Ito K., Kabeya R. (2021). Carotid endarterectomy requiring removal of the superior horn of thyroid cartilage: Case report and literature review. NMC Case Rep. J..

[22] Ho L.K., Bates T.R., Thompson A., Dharsono F., Prentice D. (2019). Cerebral embolism and carotid-hyoid impingement syndrome. J. Clin. Neurosci..

[23] Dao A., Karnezis S., Lan III J.S., Fujitani R.M., Saremi F. (2011). Eagle syndrome presenting with external carotid artery pseudoaneurysm. Emerg. Radiol..

[24] Mori M., Yamamoto H., Koga M., Okatsu H., Shono Y., Toyoda K., Fukuda K., Lihara K., Yamada N., Minematsu K. (2011). Hyoid bone compression—Induced repetitive occlusion and recanalization of the internal carotid artery in a patient with ipsilateral brain and retinal ischemia. Arch. Neurol..

[25] De Campos F.P.F., Kanegae M., Aiello V.D., Dos Santos Neto P.J., Gratao T.C., Silva E.S. (2018). Traumatic injury to the internal carotid artery by the hyoid bone: A rare cause of ischemic stroke. Autops. Case Rep..

[26] Handa J., Matsuda M., Handa H. (1972). Lateral position of the external carotid artery. Report of a case. Radiology.

[27] Teal J.S., Rumbaugh C.L., Bergeron R.T., Segall H.D. (1973). Lateral position of the external carotid artery: A rare anomaly?. Radiology.

[28] Kamide T., Nomura M., Tamase A., Mori K., Seki S., Kitamura Y., Nakada M. (2016). Simple classification of carotid bifurcation: Is it possible to predict twisted carotid artery during carotid endarterectomy?. Acta Neurochir..

[29] Katano H., Yamada K. (2010). Carotid endarterectomy for stenosis of twisted carotid bifurcations. World Neurosurg..

[30] Kathuria S., Gregg L., Chen J., Gandhi D. (2011). Normal cerebral arterial development and variations. Semin. Ultrasound CT MRI.

[31] Menshawi K., Mohr J., Gutierrez J. (2015). A functional perspective on the embryology and anatomy of the cerebral blood supply. J. Stroke.

[32] Sadler T.W. (2018). Langman’s Medical Embryology.

[33] Cairney J. (1924). Tortuosity of the cervical segment of the internal carotid artery. J. Anat..

[34] Shimizu Y., Kanetaka H., Kim Y.H., Okayama K., Kano M., Kikuchi M. (2005). Age-related morphological changes in the human hyoid bone. Cells Tissues Organs.

[35] Kamensky A.V., Pipinos I.I., Carson J.S., MacTaggart J.N., Baxter B.T. (2015). Age and disease-related geometric and structural remodeling of the carotid artery. J. Vasc. Surg..

[36] Miller K.W., Walker P.L., O’Halloran R.L. (1998). Age and sex-related variation in hyoid bone morphology. J. Forensic Sci..

[37] Abruzzo T.A., Geller J.I., Kimbrough D.A., Michaels S., Correa Z., Cornall K., Augsburger J.J. (2015). Adjunctive techniques for optimization of ocular hemodynamics in children undergoing ophthalmic artery infusion chemotherapy. J. Neurointerv. Surg..

